# Additive effect of bFGF and selenium on expansion and paracrine action of human amniotic fluid-derived mesenchymal stem cells

**DOI:** 10.1186/s13287-018-1058-z

**Published:** 2018-11-08

**Authors:** Junghyun Park, Jung Han Lee, Byung Sun Yoon, Eun Kyoung Jun, Gilju Lee, In Yong Kim, Seungkwon You

**Affiliations:** 1 Laboratory of Cell Function Regulation, Department of Biotechnology, College of Life Sciences and Biotechnology, Korea University, Anam-dong, Seongbuk-go, Seoul, 02841 Republic of Korea; 2 StemLab, Venture Incubation Center Korea University, Seoul, 136-701 Republic of Korea; 3 Institute of Animal Molecular Biotechnology, Korea University, Seoul, 136-701 Republic of Korea

**Keywords:** Amniotic fluid-derived mesenchymal stem cell, Selenium, bFGF, Conditioned media, Paracrine factors, Wound healing

## Abstract

**Background:**

Mesenchymal stem cell-derived conditioned medium (MSC-CM) has emerged as a promising cell-free tool for restoring degenerative diseases and treating traumatic injuries. The present study describes the effect of selenium as a reactive oxygen species (ROS) scavenger and its additive effect with basic fibroblast growth factor (bFGF) on in vitro expansion of amniotic fluid (AF)-MSCs and the paracrine actions of AF-MSC-CM as well as the associated cellular and molecular mechanisms.

**Methods:**

In this study, we obtained CM from human AF-MSCs cultured with selenium. The stemness of selenium-treated AF-MSCs was evaluated by cell growth and differentiation potential. Human fibroblasts were treated with AF-MSC-CM and analyzed for cell signaling changes. For in vivo wound healing assay, ICR mice with a full-thickness skin wound were used.

**Results:**

Selenium played a critical role in in vitro expansion of AF-MSCs through activation of the AKT-ERK1/2, Smad2, and Stat3 signaling pathways along with inactivation of GSK3β. When administered together with bFGF, it showed remarkable effect in inhibiting ROS accumulation and preserving their multipotency. Proliferation and migration of human dermal fibroblasts and in vivo wound healing were improved in the CMs derived from AF-MSCs exposed to selenium and bFGF, which was caused by the Smad2, AKT-MEK1/2-ERK, and NFκB signaling triggered by the paracrine factors of AF-MSCs, such as TGF-β, VEGF, and IL-6. Our results suggest the following: (a) supplementation of selenium in AF-MSC culture contributes to in vitro expansion and preservation of multipotency, (b) ROS accumulation causes progressive losses in proliferative and differentiation potential, (c) the separate activities of bFGF and selenium in MSCs exert an additive effect when used together, and (d) the additive combination improves the therapeutic effects of AF-MSC-derived CMs on tissue repair and regeneration.

**Conclusion:**

Antioxidants, such as selenium, should be considered as an essential supplement for eliciting the paracrine effects of MSC-CMs.

**Electronic supplementary material:**

The online version of this article (10.1186/s13287-018-1058-z) contains supplementary material, which is available to authorized users.

## Background

Wound healing encompasses a highly complex cascade of molecular and cellular processes, including cell migration, proliferation, angiogenesis, granulation tissue formation, re-epithelialization, and extracellular matrix (ECM) remodeling [1]. In this cascade of events, various growth factors and adhesive molecules affect the dermal fibroblasts while interacting with the other cells or ECM [2].

Human mesenchymal stem cells (MSCs), found in many tissues, such as the bone marrow, adipose, and amniotic fluid (AF), have been explored as a potential cell source for cell-based therapies due to their self-renewal and expansion capacities, plasticity, ability to downregulate immune and inflammatory responses, and differentiation into the functional cells of the mesenchymal lineage (e.g., osteoblasts, chondrocytes, myoblasts, and adipocytes) [3–5]. Moreover, as they can be directly obtained from patients, MSCs evade the ethical and safety issues associated with human embryonic stem cells or induced pluripotent cells. In particular, compared with MSCs derived from fetal or adult tissues, AF-MSCs exhibit higher proliferative and adhesive capacities and less immunogenicity, granting a privilege in clinical application [6]. Although uncertainty concerning the mechanisms by which MSCs promote wound healing is still widespread in the literature, previous studies have shown encouraging results in tissue regeneration utilizing MSC-derived conditioned medium (CM) [7, 8]. It has been suggested that endogenous repair and regenerative mechanisms are attributed to the paracrine factors secreted by MSCs rather than to their differentiation potential, which would allow them to replace damaged tissue [9]. In agreement with these findings, our previous studies demonstrated that AF-MSC-CM can enhance proliferation and migration of dermal fibroblasts in vitro, thereby accelerating wound healing in vivo [9–12]. Nevertheless, there are still several challenges to be overcome before clinical applications are possible, such as standardization of isolation protocols, in vitro expansion of MSCs to allow high throughput, retention of their multilineage differentiation potential, and enhancement of their survival and paracrine actions.

MSCs undergo replicative senescence accompanied by progressive loss of their proliferative capacity and differentiation potential during in vitro expansion [13]. Reactive oxygen species (ROS) are major mediators of cellular senescence during in vitro expansion and the wound healing process [14, 15]. In general, ROS-mediated senescence is modulated by endogenous antioxidants, such as glutathione, ascorbic acid, and enzymes related to ROS scavenging. If the antioxidant system fails to scavenge the ROS generated, then cellular oxidative stress frequently results in cell death via aging, membrane disruption, DNA damage, and inhibition of cell survival and proliferation [16]. In addition, ROS induce differentiation of MSCs into adipocytes, osteocytes, and chondrocytes, limiting their in vitro expansion [17, 18]. Reducing ROS levels could thus allow MSCs to maintain their self-renewal capacity and undifferentiated state for long-term efficacy of MSC therapy. Selenium is well known as an effective ROS scavenger that maintains cellular redox balance through activating glutathione peroxidase [19, 20] and serving as a cofactor for glutathione synthetase [21, 22]. This antioxidant has protective capabilities, including suppression of DNA damage, prevention of apoptosis, and recovery from mitochondrial dysfunction [20, 23]. Therefore, exposure of MSCs to selenium could increase their lifespan by suppressing ROS-mediated senescence and improve their paracrine effects on wound healing and tissue regeneration.

In the present study, the effect of selenium on in vitro expansion of AF-MSCs was compared with that of basic fibroblast growth factor (bFGF also known as FGF-2) alone or in combination. Although the mechanism of bFGF-induced proliferation and maintenance of differentiation potential is still a subject of debate, this growth factor has been used to extend the lifespan of MSCs in many studies [24–27]. This study thus focused on comparing selenium with bFGF in in vitro expansion and retention of multipotency of AF-MSCs and the paracrine effects of AF-MSC-CM on the in vitro proliferation and migration of human dermal fibroblasts and in vivo tissue repair and regeneration. The related molecular studies were performed to provide information on the in vitro expansion of MSCs and therapeutic efficacy of MSC-CMs and to contribute to a better understanding of their paracrine mechanisms.

## Methods

### Isolation and culture of AF-MSCs

Informed consent was acquired from three subjects, and all experiments were conducted with strict adherence to the guidelines of the Institutional Review Board of Korea University, Seoul, Korea. AF-MSCs that had been previously characterized as possessing MSC characteristics by verifying the differentiation, proliferation, and immunological phenotypes of the AF-MSCs [11, 28] were used. All experiments were performed in triplicate on independently isolated AF-MSCs. For selenium treatment experiments, three culture groups were divided as follows: AF-MSCs are cultured with low-glucose DMEM, 10% FBS, 100 U penicillin/streptomycin, 1% L-glutamine, (1) the b/− culture condition, containing 4 ng/mL bFGF (R&D Systems, Minneapolis, MN, USA); (2) the −/s culture condition, containing 5 ng/mL sodium selenite (Sigma-Aldrich, S9133), and (3) the b/s culture condition, containing 4 ng/mL bFGF and 5 ng/mL selenium. Properties of the three groups were compared, including MSC marker expression, surface antigens, and differentiation potential, as previously described [11, 28]. For preparation of CM, AF-MSCs at the 10~12 passage were plated at a concentration of 5 × 10^5^ cells/100-mm plate and incubated in proliferation medium in the presence of 4 ng/mL bFGF and/or 5 ng/mL selenium (treated with b/−, −/s, or b/s). The AF-MSCs were switched to serum-free high-glucose DMEM and incubated for three more days to generate CM. The resulting medium was collected, centrifuged at 1000 rpm for 5 min, and filtered through a 0.20-μm syringe filter as previously described [11, 28].

For growth of AF-MSCs after each treatment, AF-MSCs were cultured at a density of 3 × 10^4^ cells/well (in 12-well plates) in triplicate in growth medium with b/−, −/s, or b/s for 3 days, and then stained with 0.01% crystal violet solution. The crystal violet solution from stained cells was extracted using 10% acetic acid and subjected to spectrophotometric analysis (absorbance at 600 nm) to determine the relative cell growth rates. The differentiation potential of each AF-MSC group was evaluated by adipogenic, osteogenic, and chondrogenic differentiation according to previously reported protocols [11, 28].

### Adipogenic differentiation

Differentiation of each sample was performed according to a previously described protocol [29, 30]. Briefly, the cells were seeded at a density of 3 × 10^4^ cells/well in 6-well culture dishes and cultured in low-glucose DMEM with 10% FBS until they reached 100% confluence. They were then subjected to three cycles of induction/maintenance by sequentially culturing the cells in adipogenic induction medium [high-glucose DMEM (Invitrogen, Carlsbad, CA, USA) supplemented with 1 mM dexamethasone (Sigma-Aldrich, St. Louis, MO, USA), 0.5 mM 3-isobutyl-1-methyl-xanthine (Sigma-Aldrich), 10 ng/mL recombinant human insulin (Sigma-Aldrich), 100 mM indomethacin (Sigma-Aldrich), and 10% FBS] for 7 days, adipogenic maintenance medium (high-glucose DMEM supplemented with 10 ng/mL recombinant human insulin and 10% FBS) for 14 days, and control medium (high-glucose DMEM supplemented with 10% FBS) for 7 days. This process was repeated three times. After differentiation, the cells were fixed with 10% formalin (Sigma-Aldrich), washed, and stained with 2% (*w*/*v*) Oil Red O reagent (Sigma-Aldrich) for 5 min at room temperature to examine the generation of oil droplets in the cytoplasm.

### Osteogenic differentiation

Differentiation of each sample was performed according to a previously described protocol [29, 30]. Briefly, cells were seeded at a density of 3 × 10^4^ cells/cm^2^ in 6-well culture dishes (BD Biosciences), cultured in low-glucose DMEM (Gibco/Invitrogen) with 10% FBS until they reached 70–80% confluence, and then fed twice a week for 2.5 weeks with osteogenic induction medium [IMDM basal medium (Gibco/Invitrogen) supplemented with 100 nM dexamethasone, 10 mM β-glycerophosphate, 0.2 mM ascorbate, and 10% FBS] and control medium (IMDM basal medium supplemented with 10% FBS). Osteogenic differentiation was detected by fixing the cells with 10% formalin (Sigma-Aldrich) for 15 min at room temperature and then staining them with silver nitrate [29].

### Chondrogenic differentiation

To induce chondrogenic differentiation, cells were trypsinized, transferred into a 15-mL polypropylene tube, centrifuged at 1000 rpm for 5 min to form a pelleted micromass at the bottom of the tube, and then treated with chondrogenic medium [high-glucose DMEM supplemented with 0.1 M dexamethasone, 50 g/mL AsA (Sigma-Aldrich), 100 g/mL sodium pyruvate (Sigma-Aldrich), 40 g/mL proline (Sigma-Aldrich), 10 ng/mL TGF-β1 (R&D systems, Minneapolis, MN, USA), and 50 mg/mL ITSpremix (Gibco/Invitrogen), 6.25 μg/mL insulin, 6.25 μg/mL transferrin (Sigma-Aldrich), 6.25 ng/mL selenious acid (Sigma-Aldrich), 1.25 mg/mL bovine serum albumin (BSA), and 5.35 mg/mL linoleic acid (Sigma-Aldrich)] and control medium (high-glucose DMEM). Medium changes were carried out twice weekly, and chondrogenesis was assessed at weekly intervals. After 2–4 weeks of culture, the cells were washed twice with PBS, fixed in 4% paraformaldehyde, and visualized either by staining with Alcian Blue (Sigma-Aldrich) [11].

### BrdU assay

Cells were seeded in 4-well plates and cultured under serum-starvation conditions. After starvation, cells were cultured in triplicate under each condition in BrdU (10 μM; Sigma-Aldrich). After 24 h, cells were fixed in 4% paraformaldehyde and stained for BrdU incorporation according to the manufacturer’s instructions (Invitrogen). The BrdU label index, defined as the proportion of total cells incorporating BrdU into the nucleus, was calculated by counting BrdU-immunolabeled cells per total cells under phase contrast. The percentage of BrdU incorporation was calculated by the following formula: % of BrdU-positive cells = (number of BrdU-positive cells/number of cells) × 100%.

### RT-PCR and quantitative real-time polymerase chain reaction

RNA was prepared using TRIzol according to the manufacturer’s instructions (Invitrogen), and the cDNA was synthesized using Reverse Transcriptase II (RT, Invitrogen). To amplify the marker genes, 25 ng of cDNA was mixed along with each of the PCR primers (Bioneer, Daejeon, Korea). The PCR amplification program consisted of 24–30 cycles of the following process: melting at 94 °C for 30 s, annealing at 62 °C for 30 s, and extension at 72 °C for 30 s, followed by a final amplification step for 10 min at 72 °C. The levels of the different target markers generated by 30 PCR cycles were confirmed as being in the linear range [31]. RT-PCR was carried out using the iCycler IQ (Bio-Rad, Hercules, CA, USA). SYBR-Green PCR Master Mix (Bio-Rad, Hercules, CA, USA) was used for the chain reactions. As an internal control, the expression levels of glyceraldehyde-3-phosphatedehydrogenase (GAPDH) were quantified in parallel with those of the target marker genes. The sequences of the primers for the genes tested are listed in Additional file 1. Fold change was calculated using the ΔΔCt method [32].

### Western blotting

To analyze the changes in protein expression in response to bFGF and selenium, cells were plated at a density of 3 × 10^5^ cells/100 mm plate, allowed to attach for 12 h, and cultured with serum-free DMEM for 48 h. Cells were treated with bFGF and selenium for the indicated times (30 min–8 h). To detect protein expression of differentiated AF-MSC and changes in response to treatment with inhibitors of transforming growth factor beta (TGF-β) (SB505124), phosphatidylinositol 3-kinase (PI3K, LY294002, Sigma-Aldrich), and NF-κB (Bay-11, Santa Cruz Tech, 19542-67-7), cells were plated at a density of 7 × 10^5^ cells/100-mm plate and cultured for 30 min in the presence or absence of the inhibitors. Total protein was extracted with RIPA buffer, and the resulting lysates were centrifuged at 12,000×*g* for 30 min at 4 °C. Protein concentrations were determined using the Bradford assay kit (Bio-Rad, Hercules, CA, USA). Proteins were separated using precast 4–12% gradient SDS-PAGE (Invitrogen) and then transferred onto polyvinylidene difluoride membranes (Millipore, Bedford, MA, USA). Blots were incubated with the indicated primary antibodies at 4 °C and horseradish peroxidase-conjugated anti-mouse and anti-rabbit secondary antibodies (1:1000 dilution) at room temperature. The primary antibodies used are listed in Additional file 2, each of which was used at a final concentration of 1 μg/mL. Blots were visualized using a chemiluminescence detection system according to the manufacturer’s instructions (ECL kit; Pierce, Rockford, IL, USA). Western blot results were quantified using ImageJ software (https://imagej.nih.gov/ij/); protein expression was normalized to α-tubulin, and the ratio to relevant control was presented under individual blots as fold changes.

### FACS analysis

FACS analysis of each sample was performed according to a previously described protocol [29]. Briefly, AF-MSCs were trypsinized and transferred into FACS tubes at a concentration of 1 × 10^6^ cells/tube (BD Biosciences Clontech, Palo Alto, CA, USA). After being rinsed twice with cold buffer solution [DPBS with 1% BSA (Sigma-Aldrich, St. Louis, MO, USA) and 0.02% sodium azide, pH 7.4], the cells were incubated at 4 °C for 1 h with a primary antibody (CD13, CD14, CD15, CD29, CD31, CD33, CD34, CD44, CD45, CD71, CD90, CD106, CD117, CD120a, CD133; BD Biosciences). After incubation, the cells were washed twice with 1% BSA in PBS, resuspended in 100 μL of a fluorescein isothiocyanate (FITC)–labeled secondary antibody (diluted 1:100 in PBS with 1% BSA), and incubated for an additional 40 min at 4 °C. The cells were then washed twice with 1% BSA in PBS and fixed with a fixative solution (0.2% glucose, 2.5% formalin, and 0.02% sodium azide) in PBS for FACS analysis. To identify nonspecific signals, the control cells were incubated with isotype-matched immunoglobulins.

### ROS analysis

DHE (Invitrogen, Carlsbad, CA, USA), an oxidative fluorescent dye, was used to detect superoxide (O_2_^−^), which binds to DNA in the nucleus and fluoresces red. Briefly, AF-MSCs were trypsinized and treated with 10 μM DHE for 30 min at 37 °C in an incubator protected from light. The cells were then washed with PBS and fixed with 4% formalin in PBS for FACS analysis.

### ELISA

The paracrine factors in the AF-MSC-CMs (con, −/s, b/−, and b/s) were determined by ELISA (RayBiotech Inc., Norcross, GA, USA) according to the manufacturer’s instructions. The concentrations of TGF-β, VEGF, and IL-6 were measured using a chemiluminescence reader at 450 nm. Delta values were normalized by the extinction of the standard curves, and protein contents were calculated for each condition. Cytokine amounts were calculated in units of picograms per milliliter of medium.

### In vitro wound healing assay

In vitro wound healing assays were performed as previously described [11, 28]. Briefly, human dermal fibroblasts were seeded into 6-well dishes at a density of 7 × 10^5^ cells/well, cultured as confluent monolayers, and then synchronized in high-glucose DMEM containing 10% FBS for at least 5 h. After scratching the monolayer once with a 200-μL pipette tip, in vitro injury was induced by creating linear scratches of 500-μm-wide strips of cells. The scratch border was marked with a fine black line immediately after the scraping. The wounded cells were incubated in the presence of CM (con, b/−, −/s, or b/s) for 6–24 h. For the loss-of-function experiment, cells were treated with the inhibitors (5 μM SB505124, 50 μM LY294002, 5 μM Bay-11) for 12 h. Cell migration was assessed as a function of how far from the scratch line the cells had progressed and the overall number of cells migrating over the 18-h period.

### In vivo wound healing assay

For animal study, all procedures were approved by the Institutional Animal Care and Use Committee of Korea University (KUIACUC-2017-75). ICR mice (8 weeks old; female; body weight, 20 g) were obtained from The Daehan Bio Laboratory. For the in vivo wound healing assay, they were randomly divided into four groups (10 mice/group), and a skin wound model was generated as described previously [9, 33]. Briefly, after plucking hair from the dorsal surface and anesthetizing the mice, two 8-mm full thickness excisional skin wounds were created on each side of the midline using a punch. Each CM (con, b/−, −/s, or b/s) was topically applied to the induced wounds in a volume of 20 μL. A quick-bonding adhesive was used to fix the splint to the skin, followed by interrupted sutures to stabilize its position and the application of Tegaderm (3 M, St. Paul, MN, USA) over the wounds. Digital photographs of the wounds were taken at 0, 4, 6, and 8 days. The time to wound closure was defined as the time at which the wound was completely re-epithelialized and filled with new tissue. The wound area was measured by tracing its margin and calculated using an image analysis program (NIH Image). The percentage of wound closure was calculated as follows: (area of the original wound − area of the actual wound)/area of the original wound × 100%. The percentage of conditioned medium-induced wound closure was calculated by normalizing the value of the experimental group to the value of the CM (con)-treated group and converting the result to a percentage. Mice were sacrificed on the eighth day, and skin samples, including the wounds, were harvested for histology using a 2-mm biopsy punch. Some of the tissue specimens were fixed in 10% freshly prepared formalin for 24 h and embedded in paraffin for hematoxylin and eosin (H&E) staining [33]. Other tissue specimens were fixed in 4% paraformaldehyde for immunofluorescence analysis and 3,3′-diaminobenzidine tetrahydrochloride (DAB) staining. Four-micron-thick tissue sections were cut using a microtome and collected on Super Frost Plus poly(L-lysine)-coated slides. The embedded tissue sections were dehydrated with an ethanol gradient (70%, 80%, 95%, and 100%) and, after washing with xylene, mounted in permount solution (Fisher Scientific, Springfield, NJ, USA). The resulting paraformaldehyde-fixed tissue sections were incubated with 3% H_2_O_2_ for 10 min to block endogenous peroxidase activity and then incubated again for 60 min at room temperature with a blocking antibody and subsequently for 16–18 h at 4 °C with the primary antibodies (anti-pAKT, anti-pERK, anti-pSMAD2/3, anti-NFκb; involucrin 1:200 dilution). After being washed with PBS, the tissue sections (anti-pAKT, anti-pERK, anti-pSMAD2/3, anti-NFκb) were incubated for 1 h at room temperature with a secondary anti-mouse or anti-rabbit antibody (1:400 dilution) and again incubated for 30 min with horseradish peroxidase and DAB as the chromogen. For involucrin staining, the secondary Cy3-labeled antibodies (1:500) were treated for 2 h and washed twice with PBS. Sectioned tissues were incubated in 4,6-diamidino-2-phenylindole (DAPI) for nucleic staining. The stained sections were imaged with an Olympus DP70 camera system (Olympus, Tokyo, Japan). IHC scoring was conducted by “quick score (*Q*)”. The scores were obtained by multiplying the percentage of positive cells (*P*) by the intensity (*I*) [formula: *Q* = *P* × *I*; maximum = 300] [34]. This study was conducted in accordance with the guidelines for the care and use of laboratory animals provided by Korea University, and all experimental protocols were approved by the Ethics Committee of Korea University.

### Statistical analysis

All values are expressed as mean ± SD. Data comparisons were made using one- or two-way ANOVAs with post hoc Tukey’s tests and paired, two-tailed Student’s *t* tests. Differences with *p* < 0.01 and < 0.05 were considered statistically significant.

## Results

### In vitro expansion and intracellular ROS accumulation

**Fig. 1:**
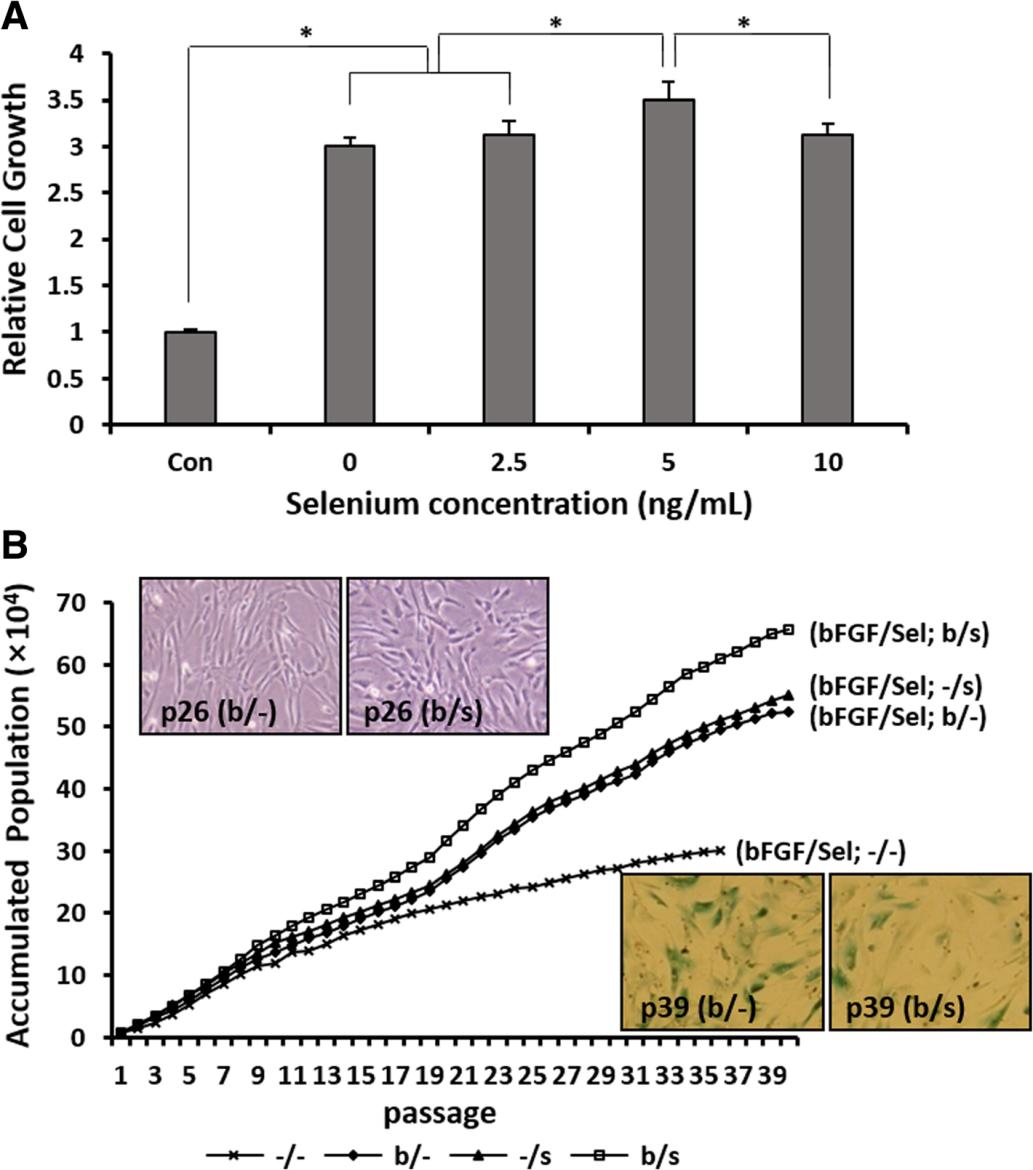
In vitro expansion of AF-MSCs treated with bFGF and selenium. **a** Relative growth rates of AF-MSCs after 3 days of exposure to various concentrations of selenium (0, 2.5, 5, and 10 ng/mL) under bFGF (4 ng/mL) supplementation. The growth rates were determined by crystal violet assay. Error bars represent the mean ± SD of three independent experiments performed in triplicate. *p < 0.05. **b** Proliferation of AF-MSCs cultured with bFGF (4 ng/mL) and selenium (5 ng/mL), alone or in combination, until they reached senescence. The inset images show the morphology of AF-MSCs in the proliferation stage (top images) and large and flattened AF-MSCs expressing β-gal as a marker of cellular senescence (bottom images), respectively

**Fig. 2:**
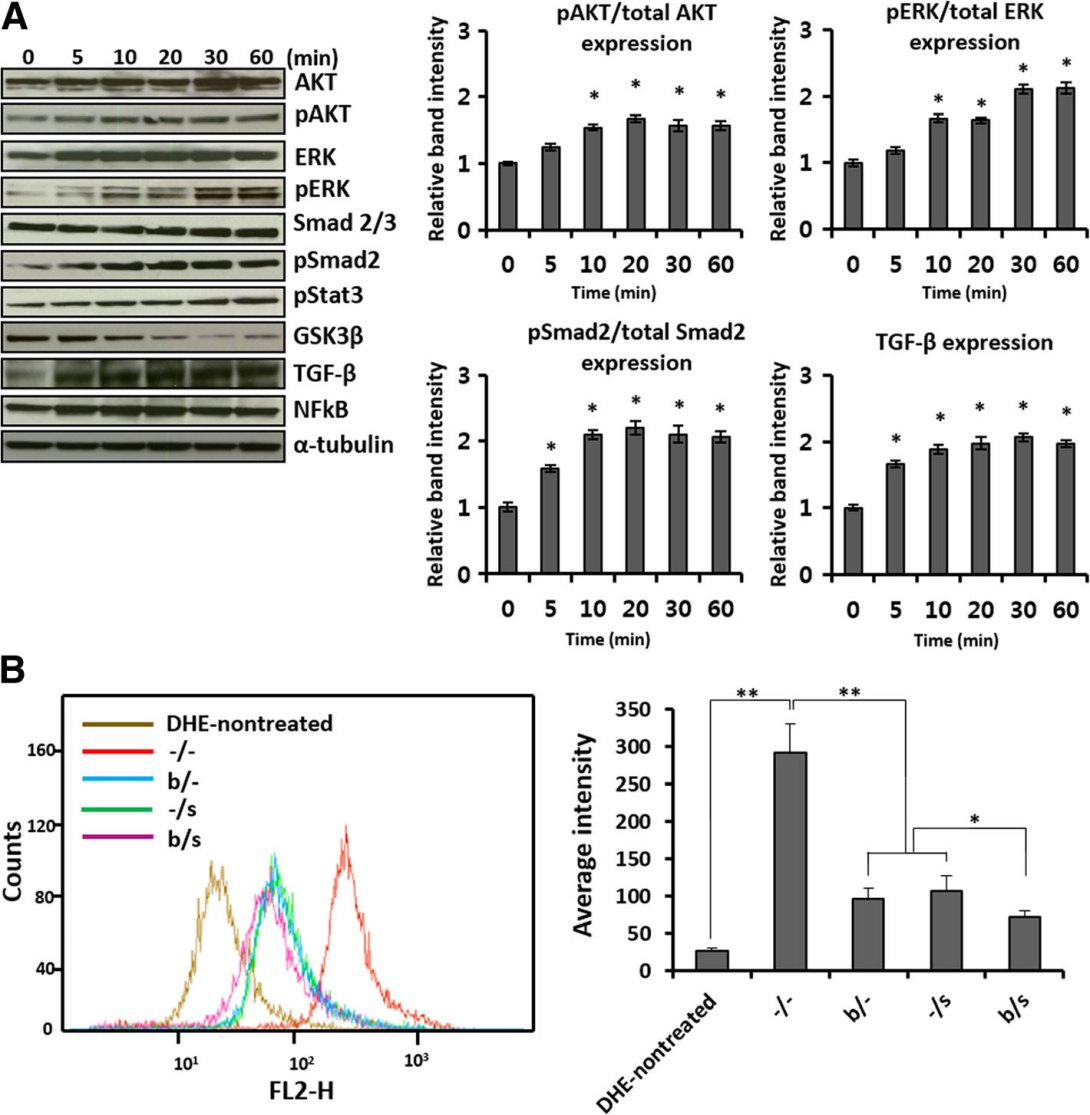
Intracellular response of AF-MSCs to selenium exposure. **a** Time-dependent changes in signaling pathways (pAKT, pERK, pSMAD2, GSK3, TFG-β, and NFκB) during exposure of AF-MSCs to selenium. Error bars represent the mean ± SD of three independent experiments performed in triplicate. **b** ROS generation in AF-MSCs cultured with bFGF (4 ng/mL) and selenium (5 ng/mL), alone or in combination, for two passages. The ROS levels were determined by DHE staining and flow cytometry. Data are represented as mean ± SD (*n* = 5). *p < 0.05, **p < 0.01

To determine the optimal concentration of selenium, we compared the growth rate of AF-MSCs cultured with various concentrations of selenium (0, 2.5, 5, and 10 ng/mL) under bFGF supplementation. The chosen concentration (4 ng/mL) of bFGF was based on the culture conditions of stem cells previously described [11, 35]. Figure 1a shows the additive effect of selenium and bFGF on the growth of AF-MSCs during 3 days of culture. The highest growth rates were observed in the AF-MSCs incubated with 5 ng/mL selenium under bFGF supplementation; thus, this selenium dose was chosen as an optimal concentration for subsequent studies. During extended culture of AF-MSCs, their proliferation rates were gradually decreased, and they began to undergo senescence after 38–40 passages (120–144 days in culture) (Fig. 1b). It has been widely accepted that a slow proliferation after the phase of near-linear growth indicates a decline of proliferation capacity and further cellular senescence, as described by Hayflick [36]. The optical images show the morphology of AF-MSCs during proliferation (top layer) and large and flattened AF-MSCs expressing β-gal as a marker of cellular senescence (bottom layer). The proliferation rate of AF-MSCs was significantly higher after bFGF and selenium (b/s) treatment than after treatment with bFGF alone (b/−) or selenium alone (−/s), suggesting that the proliferative capacity of MSCs can be improved by the additive combination of bFGF and selenium (Fig. 1b). Furthermore, possible signaling pathways involved in the improvement of AF-MSC proliferation during selenium exposure were identified. Figure 2a indicates that selenium induced the expression of p-AKT, p-ERK, p-Smad2, and TGF-β while reducing the expression of GSK3β as a function of time, which occurred immediately after exposure of AF-MSCs to selenium. On the other hand, at the end of their extended lifespan, chromosome analysis was performed using G-banding to assess chromosomal abnormalities leading to genetic disorders and malignancy. Additional file 3 shows a normal karyotype of the human AF-MSCs treated with bFGF and selenium, either alone or in combination.

Intracellular ROS levels were measured to evaluate the contribution of selenium as a ROS scavenger to the improved proliferation of AF-MSCs. As shown in Fig. 2b, significant differences were observed in the ROS levels depending on the presence or absence of bFGF and selenium, and further, the combination of these treatments resulted in the greatest reduction in ROS levels. As a result, the findings demonstrated that selenium induces proliferation of AF-MSCs through activation of the signaling pathways related to stimulation of cell proliferation and, concurrently, inhibition of GSK3β and ROS levels.

### Preservation of phenotype and multipotency

**Fig. 3:**
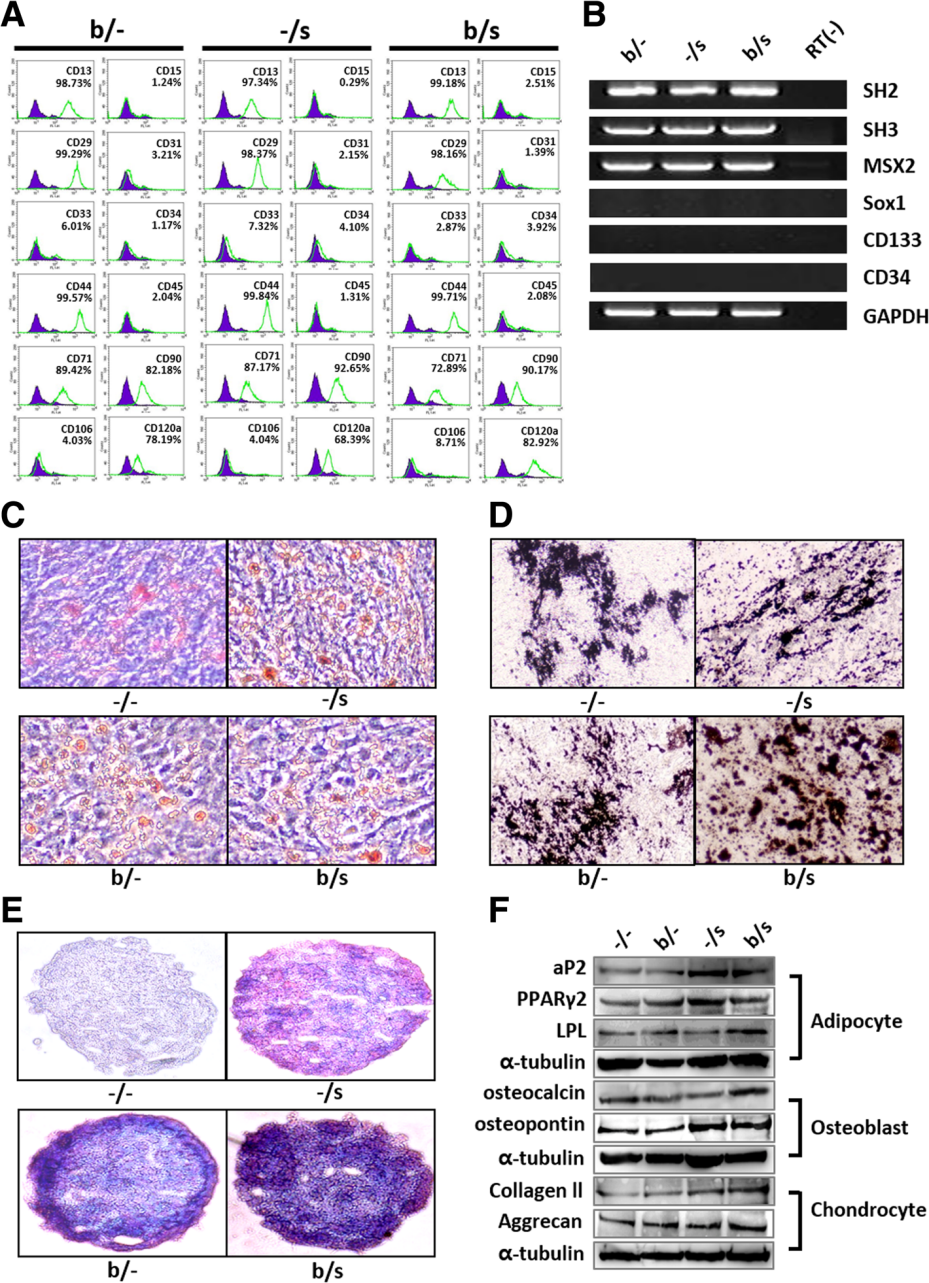
Retention of phenotype and multipotency of AF-MSCs during bFGF and selenium treatments. **a** Flow cytometry analysis of AF-MSC surface markers. The MSC-specific markers CD13, CD29, CD44, CD71, CD90, and CD120a were observed, while the endothelial cell (CD31, CD106) and hematopoietic (CD15, CD33, CD34, CD45) markers were not expressed. **b** mRNA expression analysis of markers specific to MSCs and other cell types, such as neural stem cells (Musashi1, Sox1) and hematopoietic cells (CD133, CD34). **c** Adipogenic differentiation of AF-MSCs after 3 weeks of exposure to bFGF and selenium, alone or in combination (−/s, b/−, or b/s). **d** Osteogenic differentiation of AF-MSCs after 3 weeks of exposure to bFGF and selenium. **e** Chondrogenic differentiation of AF-MSCs after 3 weeks of exposure to bFGF and selenium. The differentiation of AF-MSCs into the three lineages was evaluated by Oil Red O, Von Kossa, and Alcian Blue staining. **f** Protein expression analysis of adipogenic, osteogenic, and chondrogenic markers in the differentiated AF-MSCs

To assess whether AF-MSCs cultured in the presence of bFGF and selenium, alone or in combination, maintain their stem cell characteristics, their surface markers and multipotency were investigated. Figure 3a shows that AF-MSCs treated with bFGF and/or selenium expressed typical MSC marker proteins, such as CD13, CD29, CD44, CD71, CD90, and CD120a. The endothelial (CD31 and CD106) and hematopoietic markers (CD15, CD33, CD34, and CD45) were not expressed as the isotype control. Subsequently, mRNA expression levels of AF-MSC markers and the markers of other cell lineages (e.g., neural stem cells and hematopoietic cells) were analyzed by RT-PCR. The results indicate that the identity of the AF-MSCs was maintained during selenium treatment (Fig. 3b). The multipotency of AF-MSCs treated with bFGF and selenium was evaluated by their differentiation into adipocytes, osteoblasts, and chondrocytes in vitro. Figure 3c–e shows optical images of adipogenic, osteogenic, and chondrogenic differentiation of the AF-MSCs (10 passages), respectively, cultured in the presence of bFGF and selenium, alone or in combination. Although there were no significant differences between the culture conditions, the multipotency of AF-MSCs was preserved during their in vitro expansion. This observation was supported by the expression of markers specific to adipogenesis (aP2, PPARγ2, and LPL), osteogenesis (osteocalcin and osteopontin), and chondrogenesis (type II collagen and aggrecan) (Fig. 3f). These results indicate that exposure of AF-MSCs to selenium can improve the proliferation and extend the lifespan of AF-MSCs, preserving their multipotency.

### In vitro evaluation of AF-MSC-CMs and secretome profiling

**Fig. 4:**
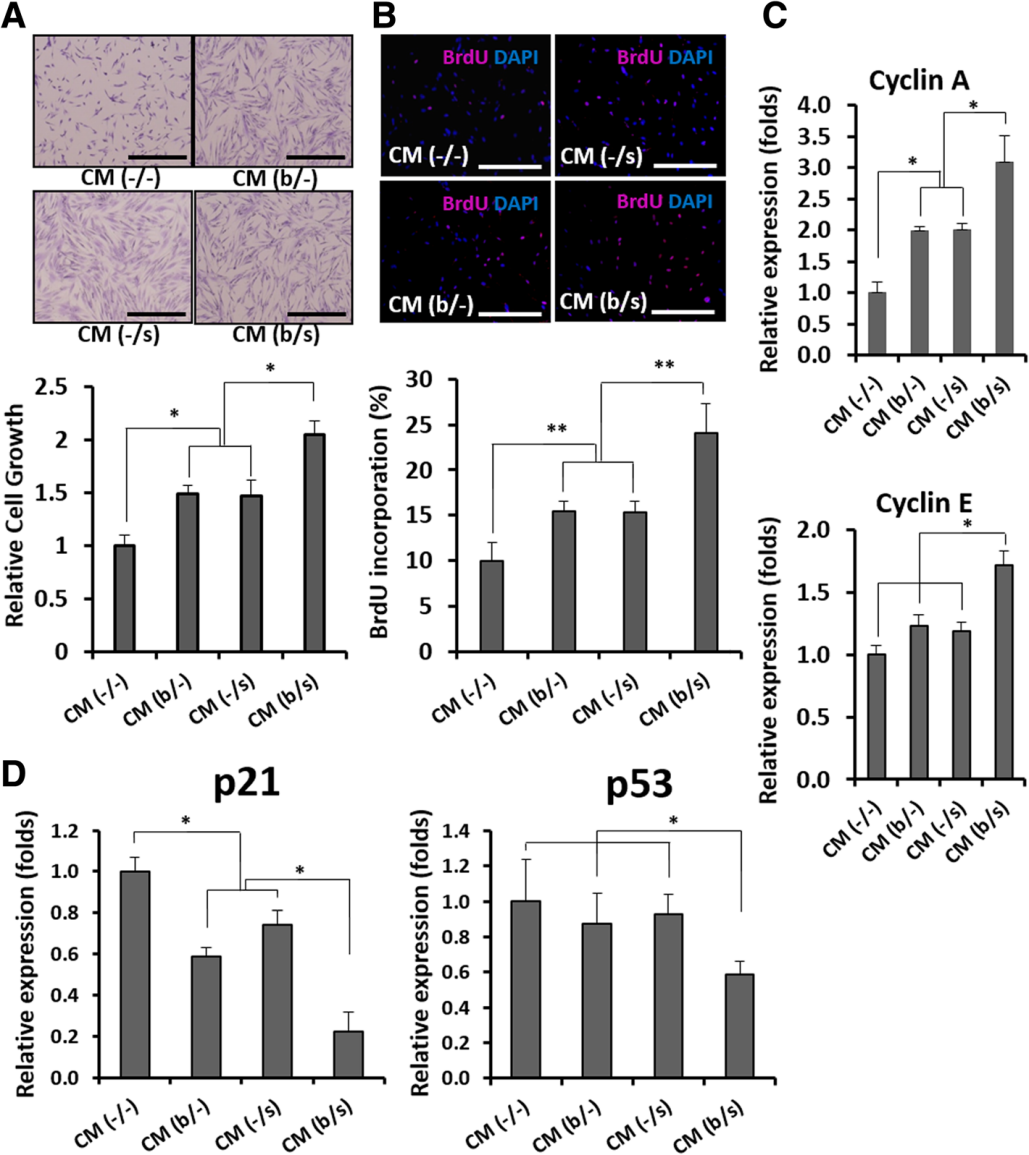
Proliferation of human dermal fibroblasts cultured in the AF-MSC-CMs. **a** Optical images and relative growth rates of dermal fibroblasts after 3 days of culture in AF-MSC-CM (b/−), CM (−/s), and CM (b/s) obtained from AF-MSCs cultured with bFGF, selenium, or both, respectively. Scale bars, 1 mm. **b** Fluorescence images and relative quantification of BrdU-labeled AF-MSCs after 3 days of culture in AF-MSC-CM (b/−), CM (−/s), and CM (b/s). Scale bars, 1 mm. **c** Cell cycle progression of human dermal fibroblasts induced by AF-MSC-CM. **d** Senescence-associated mRNA expression level of human dermal fibroblasts induced by AF-MSC-CM. Error bars represent the mean ± SD of three independent experiments performed in triplicate. *p < 0.05, **p < 0.01

**Fig. 5:**
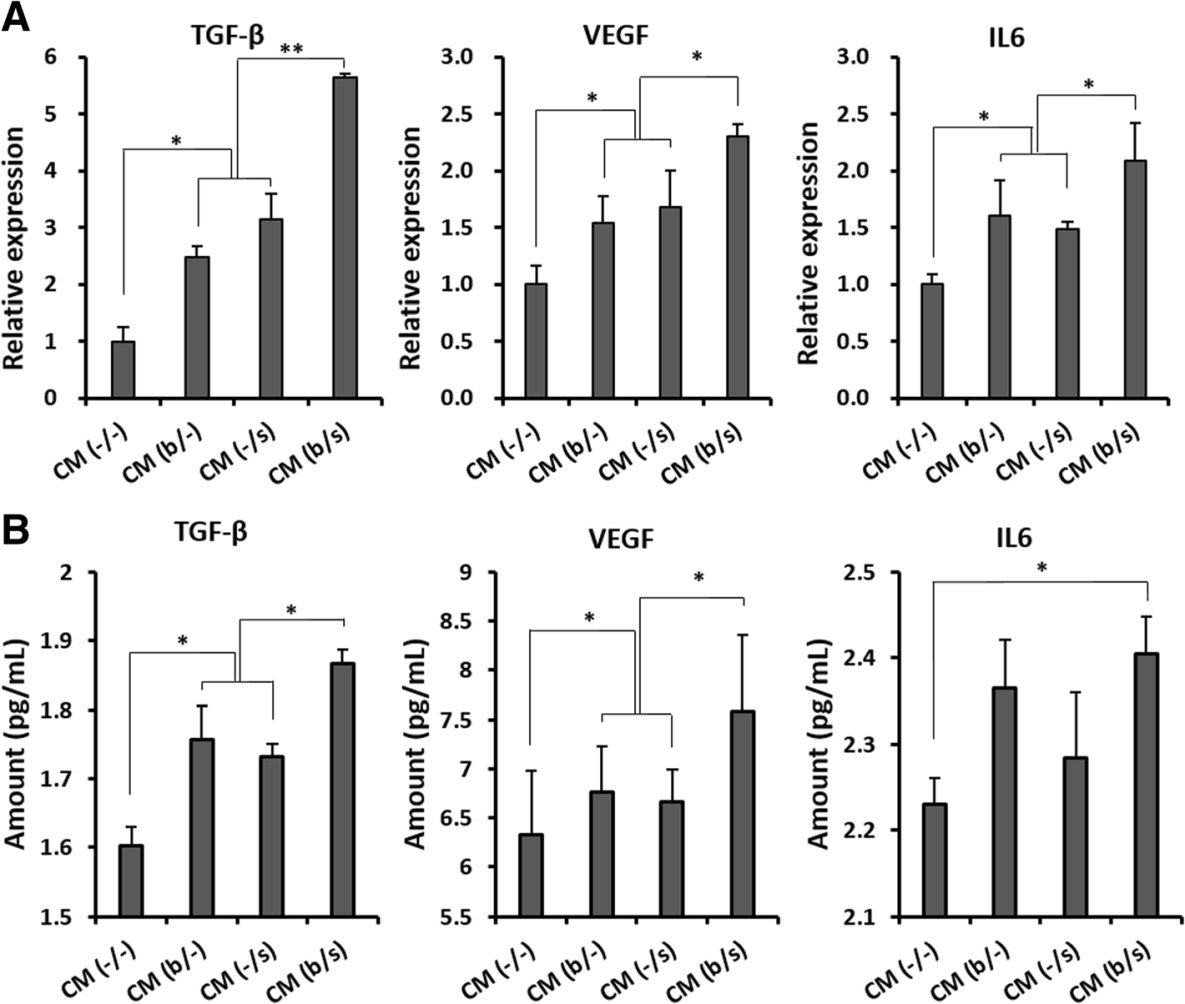
Production of paracrine factors by AF-MSCs. **a** Relative mRNA expression and **b** protein levels of the corresponding paracrine factors (TGF-β, VEGF, and IL-6) secreted by AF-MSCs treated with bFGF and/or selenium. Error bars represent the mean ± SD of six independent experiments performed in triplicate. *p < 0.05, **p < 0.01

**Fig. 6:**
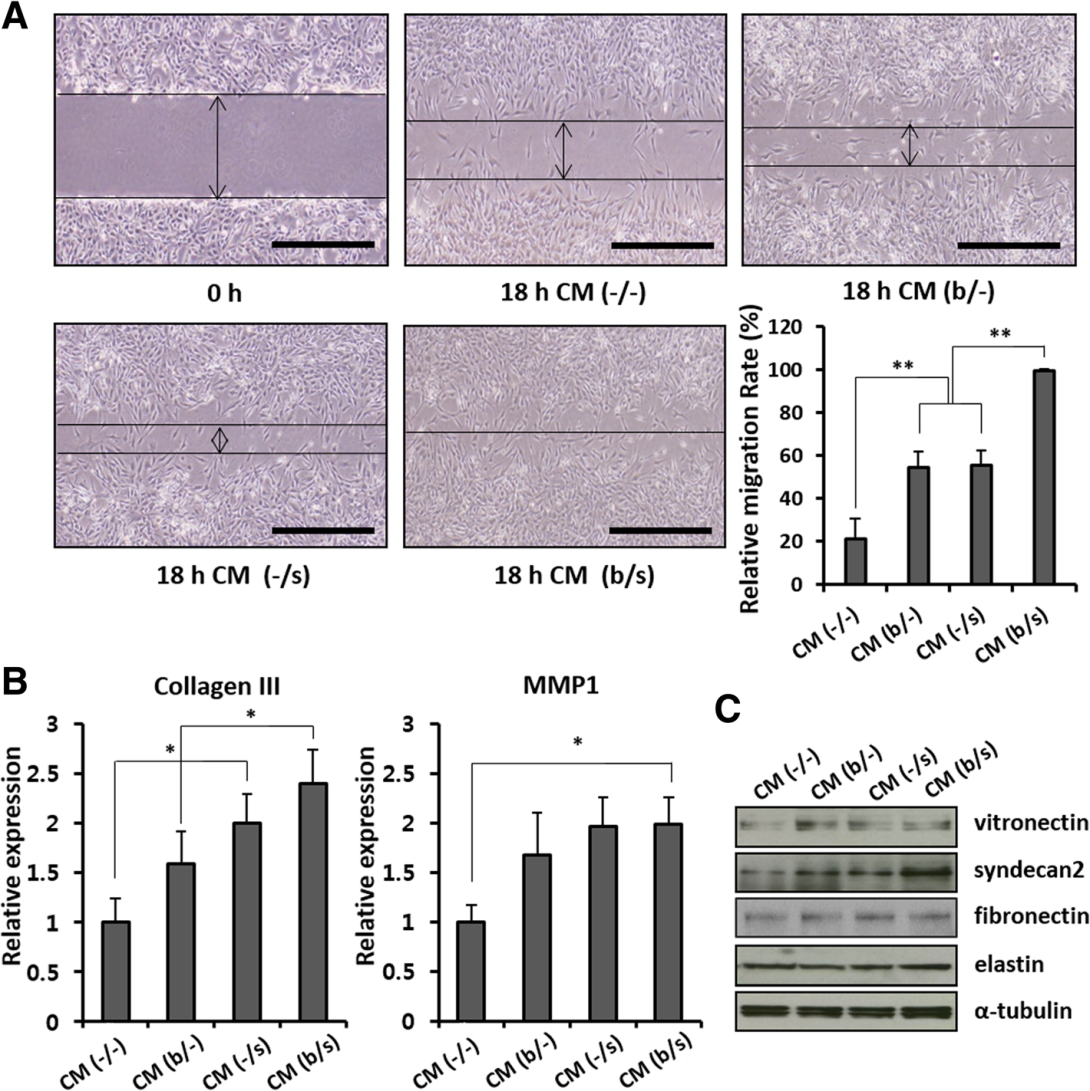
Migration behavior of human dermal fibroblasts in AF-MSC-CM (b/−), CM (−s), and CM (b/s). **a** Optical images of dermal fibroblasts taken from the scratch-wound-closure assay and their relative migration rates. Dermal fibroblasts were synchronized by serum starvation, wounded, and cultured for 18 h. Arrows indicate the wound size. Scale bars, 1 mm. **b** Relative quantification of ECM molecules (collagen III and MMP1) secreted during dermal fibroblast migration. **c** Western blot comparison of expression levels of migration factors (vitronectin, syndecan 2, fibronectin, and elastin) in dermal fibroblasts. Error bars represent the mean ± SD of three independent experiments performed in triplicate. *p < 0.05, **p < 0.01

In an effort to evaluate the effect of AF-MSC-CMs on cell proliferation and migration and to understand the relevant mechanisms, human dermal fibroblasts were cultured in vitro in the CM (b/−), CM (−/s), or CM (b/s) from the AF-MSCs treated with bFGF and/or selenium. Dermal fibroblasts cultured with CM (−/−) served as a control. Figure 4a, b shows the optical and BrdU fluorescence images and relative growth rates of dermal fibroblasts after 3 days of culture in the CMs. Their proliferation significantly increased in all the CMs and is the highest in CM (b/s), which is well supported by comparing expressed cyclin A and E and p53/p21 (Fig. 4c, d). The fibroblasts cultured with CM (b/−) and (−/s) showed no significant difference in cell cycle progression while exhibiting the most vigorous cell division in CM (b/s). Based on the results, it appears that treatment with bFGF and/or selenium stimulates the production of paracrine factors from AF-MSCs, which promote the wound healing process. As expected, the mRNA levels of transforming growth factor beta (TGF-β), vascular endothelial growth factor (VEGF), and interleukin-6 (IL-6) were elevated in the AF-MSCs cultured with bFGF and/or selenium (Fig. 5a). The concentration of the corresponding proteins was higher in the CM (b/s) (Fig. 5b), indicating the beneficial effects of bFGF and selenium on wound healing. Furthermore, the paracrine effects of AF-MSC-CMs on in vitro migration of dermal fibroblasts were examined. Figure 6a clearly shows that dermal fibroblasts migrated most actively in CM (b/s) culture. We also found relatively higher levels of migration-associated factors, such as matrix metalloproteinase 1 (MMP1), collagen III, vitronectin, syndecan 2, fibronectin, and elastin, in the CMs derived from bFGF and/or selenium-treated AF-MSCs (Fig. 6b, c).

**Fig. 7:**
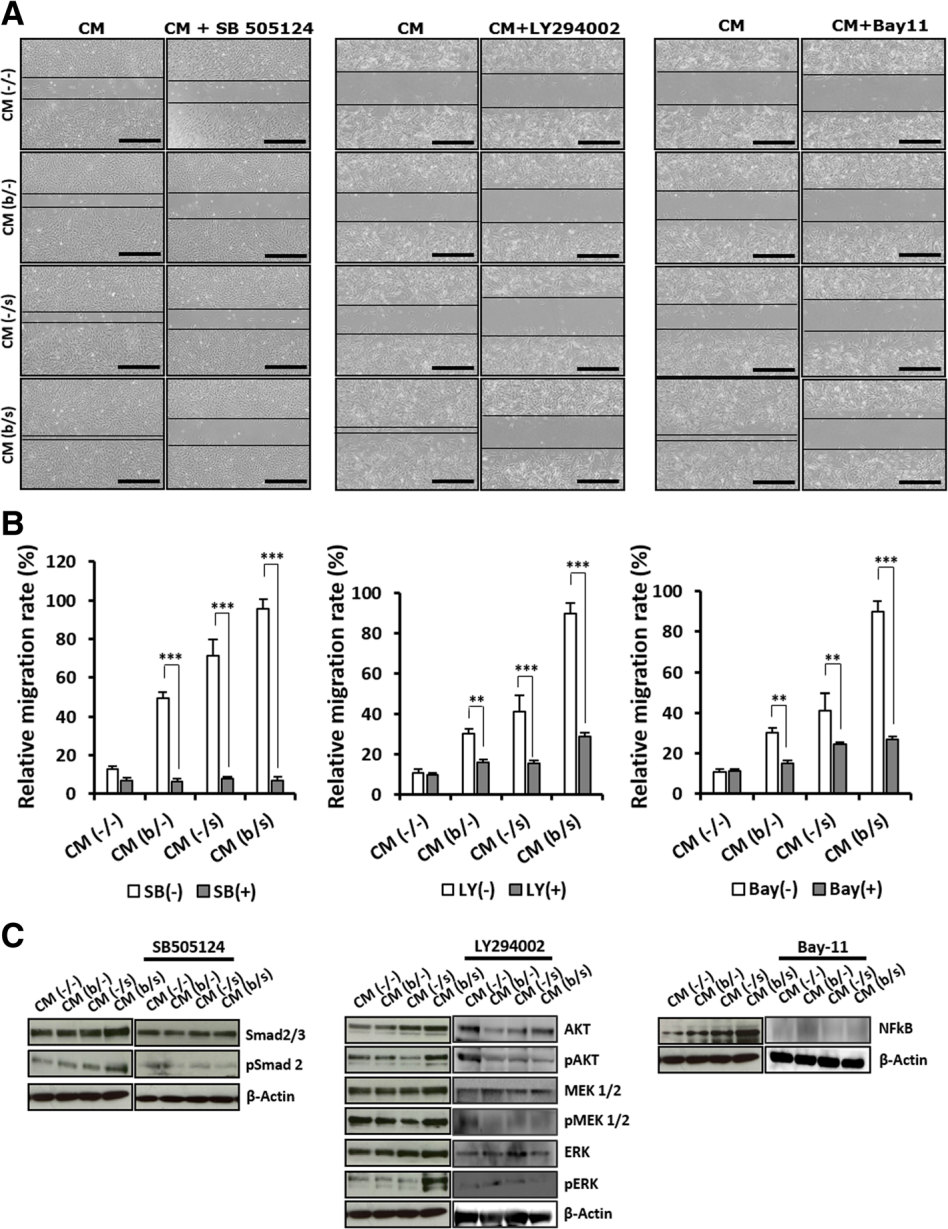
Molecular mechanism of human dermal fibroblast migration in AF-MSC-CM (b/s). **a** Optical images (scale bars, 1 mm) and **b** relative migration rates of dermal fibroblasts after 18 h of culture in AF-MSC-CM (b/s) untreated (CM) and treated with SB5050124, LY294002, or Bay11. SB5050124, LY294002, and Bay11 are selective inhibitors of the TGF-β, PI3K, and NFκB cell signaling pathways, respectively. **c** Western blot comparison of phosphorylated signaling molecules in response to SB5050124, LY294002, and Bay11 in dermal fibroblasts. The migration of dermal fibroblasts was improved by the signaling pathways depending on Smad2, AKT-MEK1/2-ERK, and NFκB. Error bars represent the mean ± SD of three independent experiments performed in triplicate. The asterisk denotes a statistically significant difference between the migration rates of untreated dermal fibroblasts and those treated with SB5050124, LY294002, or Bay11 (*p < 0.05, **p < 0.01, and ***p < 0.001)

As correspondingly activated by TGF-β, VEGF, and IL-6 in the CM of AF-MSCs treated with bFGF and selenium, the total protein and phosphorylation levels of Smad2, AKT-MEK1/2-ERK, and NFκB were investigated as the signaling molecules involved in the migration of dermal fibroblasts cultured in AF-MSC-CMs (Fig. 7). All the CMs induced activation of signaling molecules such as Smad2, AKT-MEK1/2-ERK, and NFκB, thereby leading to dermal fibroblast migration. While there was no significant difference in the migration of cells treated with CM (b/−) or CM (−/s), CM (b/s) resulted in the greatest migration behavior of dermal fibroblasts. Furthermore, for a better understanding of the signaling molecules involved in migration, inhibitors of Smad2, AKT-MEK1/2-ERK, and NFκB (SB505124, LY294002, and Bay11, respectively) were added to the AF-MSC-CMs. As shown in Fig. 7b, c, when the signaling of these molecules was reduced by the inhibitors, minimal or no migration of dermal fibroblasts was observed in all of the groups. These results suggest that cell proliferation and migration were improved by the signaling pathways depending on Smad2, AKT-MEK1/2-ERK, and NFκB, which were activated by TGF-β, VEGF, and IL-6, respectively.

### In vivo evaluation of AF-MSC-CMs

**Fig. 8:**
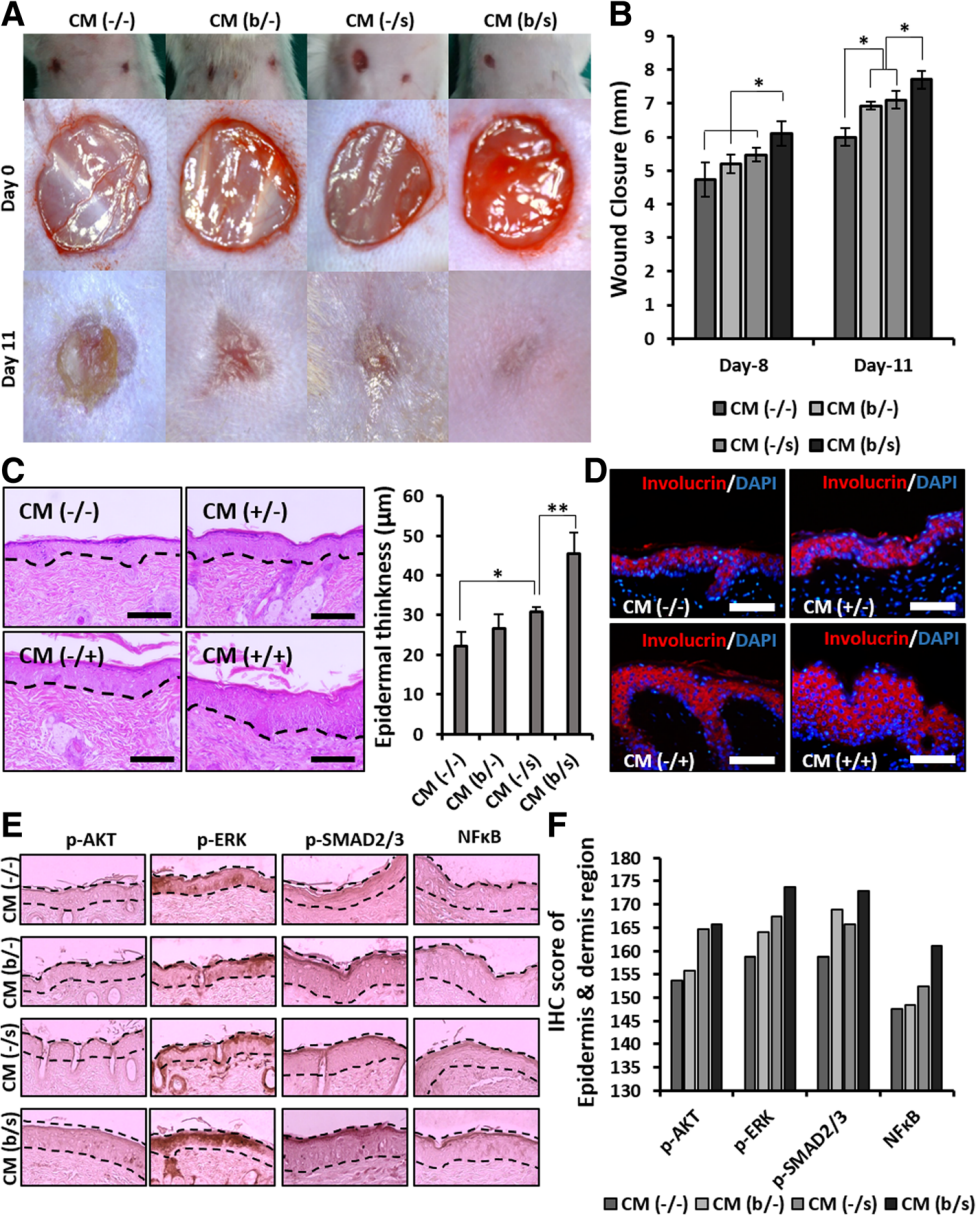
Paracrine effects of AF-MSC-CM (b/−), CM (−/s), and CM (b/s) on skin wound healing. **a** Representative images of wounds after 11 days of treatment with AF-MSC-CM (b/s). **b** Measurement of wound size. Error bars represent the mean ± SD of three independent experiments performed in triplicate. *p < 0.05. **c** H&E stained histological images of mouse back skin. Thickness means of epidermis were obtained from 10 randomly chosen sites. *p < 0.05, **p < 0.01. Scale bars, 50 μm. **d** Fluorescence images of involucrin expression (marker of epidermal keratinocyte) in epidermis. Scale bars, 50 μm. **e** Immunohistochemical analysis of AKT, ERK, SMAD2/3, and NFκB in wounded skin. The black dashed line indicates the epidermal and dermal regions. **f** Quantification of DAB staining in the epidermal and dermal regions by IHC scoring. High positive, + 3; positive, + 2; trace negative, + 1; negative, 0. IHC scoring was conducted by “quick score (*Q*)”. The scores were obtained by multiplying the percentage of positive cells (*P*) by the intensity (*I*) [formula: *Q* = *P* × *I*; maximum = 300]

To test the feasibility of employing the AF-MSC-CMs, in vivo wound healing study was performed on the skin of imprinting control region (ICR) mice. Wounds applied by vehicle medium (CM(−/−)) were used as a control. Treatment with CM (b/s) resulted in complete wound closure after 11 days, indicating that the additive combination of bFGF and selenium accelerated cell proliferation and migration during wound healing in comparison with the other groups (Fig. 8a, b). Interestingly, the CM (−/s) group exhibited better recovery than the CM (b/−) group, which correlated well with the results of the in vitro proliferation and migration of human dermal fibroblasts. Likewise, in re-epithelialization assessment using H&E staining and involucrin immunofluorescence, the CM (b/s) group showed the thickest epidermis region and the highest expression of involucrin (epidermal marker) among the groups (Fig. 8c, d). Furthermore, an analysis of immunohistochemistry (IHC) scores demonstrated that the Smad2, AKT-MEK1/2-ERK, and NFκB signaling pathways were more effectively activated by CM (−/s) during the wound healing process than by CM (b/−), and their highest activation was seen when treated with CM from MSCs administered selenium in combination with bFGF (Fig. 8e, f).

## Discussion

In vitro expansion, long-term survival and function of MSCs, and preservation of their multipotent differentiation capacity are important prerequisites for the application of MSC-derived CM to wound healing and tissue regeneration. Since bFGF, as one of the growth factors involved in wound healing, has been widely used in many studies demonstrating the proliferation and differentiation potential of MSCs during in vitro expansion [24, 26, 37], it was chosen as a reference supplement for evaluation of the paracrine effects of selenium-treated AF-MSCs. The present study showed that AF-MSCs treated with bFGF allowed improved proliferation and lifespan (Fig. 1a, b), which was in agreement with the previous studies that reported the Hayflick limitation [27, 38]. By contrast, Yanada et al. reported that MSCs treated with bFGF could undergo > 80 population doublings, exceeding the Hayflick number [26]. These conflicting results are still the subject of debate. Interestingly, with the combination of bFGF and selenium, our results showed much greater expansion, with MSCs undergoing 40 passages in approximately 144 days of in vitro culture before reaching senescence, even in comparison with previous studies that supplemented MSC culture with various growth factors and cytokines for improvement of MSC proliferation [39, 40]. In an effort to understand the additive effects of bFGF and selenium on improving AF-MSC proliferation, we first confirmed the activation of the signaling molecules contributing to proliferation in the AF-MSCs treated with selenium and subsequently compared cellular ROS levels among cells treated with bFGF and selenium, alone or in combination. The critical role of ROS in inflammatory, fibrogenic, carcinogenic, and apoptogenic processes is well established in the literature. Intracellular ROS accumulation leads to passage-dependent senescence of MSCs through the changes in the mRNA expression of genes related to mitochondria, glutathione metabolism, and antioxidant defense [41, 42]. Especially, ROS-induced mitochondrial dysfunction accelerates cellular senescence by increasing oxidative stress in normal somatic cells as well as in MSCs. This vicious cycle of senescence results in the loss of the long-term proliferative and multipotent capacity of MSCs. Our results clearly indicate that bFGF and selenium can enhance in vitro expansion of AF-MSCs by activating the AKT-ERK1/2, Smad2, and Stat3 signaling pathways, inactivating GSK3β and inhibiting ROS accumulation (Fig. 2). Intracellular signaling activation induced by bFGF has been widely explored in MSCs originated from various tissues. For instance, bFGF-triggered activation of AKT and ERK pathway promotes differentiation and proliferation in BM-MSCs [43]. Another study showed that the expression of cytokines, such as TGF-β and VEGF, is upregulated by bFGF in placenta-derived mesenchymal stem cells [44]. A significant decrease in the ROS levels was found after treatment with selenium and bFGF. The ROS levels mirror what was observed in the proliferation studies, supporting the additive effects of bFGF and selenium. This result implies that the reduction of ROS plays a pivotal role in in vitro expansion and long-term survival of MSCs. Although selenium is well known to be an effective antioxidant and free radical scavenger, only a few studies on its effects on MSCs have been recently reported and none on its effects on extending the proliferation and differentiation potential of MSCs [45–47]. Meanwhile, recent research has provided some interesting clues to explain the observed bFGF-induced ROS reduction. Based on their work with acute myocardial ischemia/reperfusion (I/R) injuries, Wang et al. reported that bFGF significantly reduces apoptosis by inhibiting ROS-induced mitochondrial dysfunction through expression of proteins related to the MAPK/ERK and PI3K/AKT signaling pathways [48]. Furthermore, related studies showed that, in the case of high-glucose-induced ROS generation, cell migration and skin regeneration are promoted by activation of the PI3K-Rac1-JNK pathway and regulation of MAPK family proteins via bFGF signaling [49–51]. This study indicated that treatment of selenium effectively changed the activation of AKT and ERK pathway. However, the mechanism of how selenium activates these signal pathways is not clear. Further studies should be performed to make clear the effect of selenium to amniotic fluid-mesenchymal stem cells.

In addition to the proliferative capability of MSCs, a supplement should only be considered as a suitable candidate for their in vitro expansion if the expanded cells retain their multipotency. In this study, for all three treatments, the AF-MSCs showed not only MSC-specific marker proteins and mRNA expression but also the ability to differentiate into adipocytes, osteoblasts, and chondrocytes. Expression of the CD120a marker protein, known as the tumor necrosis factor (TNF) receptor, which is associated with modulation of TNF-α, inducing mitochondrial ROS production following cell death, increased in AF-MSCs after treatment with a combination of bFGF and selenium [52]. Previous work showed that MSCs transduced with the TNF receptor gene showed improved survival and ventricular function after transplantation at an acute myocardial infarction site due to attenuated TNF-α levels in the serum and cardiac tissue [53]. Furthermore, multipotency of AF-MSCs was well preserved during bFGF and/or selenium culture (Fig. 3c–e). Overall, we found that bFGF and selenium allow in vitro expansion of AF-MSCs, preserving their multipotency for long-term culture, and further serve as a critical tool in regulating the intracellular ROS levels that decide cell fate. The most striking result was that the additive combination of bFGF and selenium on AF-MSCs contributed most effectively to their proliferation, ROS defense, stemness, and suppression of senescence. This synergism may be based on a combination of two separate processes: (1) bFGF-induced signaling, which stimulates self-renewing proliferation and alleviates cellular senescence, and (2) selenium-mediated antioxidant processes, which prevent ROS-dependent senescence and damage. In particular, their synergistic effect is closely associated with reduced accumulation of intracellular ROS.

MSC-mediated tissue repair and regeneration can be achieved by the cells’ proliferation and differentiation capabilities as well as their paracrine effects that provide the secreted cytokines and proteinases, such as VEGF, MMPs, TGF-β, and bFGF, required for angiogenesis and tissue remodeling [54]. Accordingly, MSC-derived CM containing such paracrine factors has emerged as a promising cell-free treatment for degenerative diseases and traumatic injuries [55]. In the present work, the remarkable additive effect of bFGF and selenium on AF-MSCs naturally led to an enhancement of paracrine potential in the AF-MSC-derived CMs. The in vitro proliferation and migration of human dermal fibroblasts were similar to those observed during the AF-MSC expansion assessment, in which, after exposure to bFGF and selenium, there were improvements of cell proliferative capacity and their additive effect to AF-MSCs’ response. These results imply that the productivity of paracrine factors secreted from AF-MSCs was stimulated by bFGF and selenium and dramatically boosted by their combination, which was proven by the elevated mRNA levels of TGF-β, VEGF, and IL-6, regarded as key paracrine factors for tissue repair (Fig. 5a) [56], and the high levels of the corresponding proteins in the AF-MSC-CMs (Fig. 5b). Our previous studies showed that the proliferation and migration of dermal fibroblasts were enhanced in AF-MSC-CM culture through PI3K/AKT and TGF-β/Smad2 signaling, thereby accelerating wound healing [11, 28]. More importantly, the present work clearly shows that the paracrine effects of AF-MSC-CM can be enhanced not only by pretreatment with bFGF and selenium but also more effectively by their synergistic combination. This observation is supported by the increased expression of migration-associated factors, including MMP1, collagen III, vitronectin, syndecan 2, fibronectin, and elastin (Fig. 5b, c). In wound healing, the most prevalent fiber-forming proteins include collagen, fibrin, fibronectin, vitronectin, elastin, and fibrillin [57]. These ECM components are mainly produced by dermal fibroblasts and simultaneously regulate cell function in some situations, which is a crucial mechanism for skin regeneration [58]. Furthermore, we found that the paracrine factors secreted by AF-MSCs exposed to bFGF and selenium triggered the AKT-MEK-ERK, Smad2/3, and NFκB signaling pathways, which are involved in the proliferation and migration of dermal fibroblasts (Fig. 7). In particular, the Smad2 inhibition by SB 505124 suggests that the signaling of TGF-β through the TGF-β/Smad2 axis plays an important role in the migration of dermal fibroblasts [59].

Many in vivo and in vitro studies using various models have investigated the involvement of TGF-β as a leading factor in inflammation, angiogenesis, re-epithelialization, and connective tissue regeneration, and its expression increases at the onset of injury [55]. Barrientos et al. reported that TGF-β promotes granulation tissue formation by increasing the expression of genes associated with ECM remodeling and upregulating VEGF expression [55]. Ironically, TGF-β increases intracellular ROS accumulation by impairing mitochondrial function and suppresses the antioxidant system, leading to oxidative stress or redox imbalance [60]. Accordingly, in AF-MSCs exposed to both bFGF and selenium, selenium might increase TGF-β secretion while the cells intensively secrete paracrine factors, including VEGF and IL-6, through bFGF- and TGF-β-induced signaling (Fig. 2a, b), supported by the relatively high levels of VEGF and IL-6 secreted from CM (−/s) (Fig. 5a, b). These factors play a vital role in tissue repair and regeneration [56, 61]. These observed preferences for treatment of bFGF and selenium in combination were directly reflected in the in vivo wound healing results (Fig. 8). Again, the injured skin recovered and regenerated the fastest in the mice treated with AF-MSC-CM (b/s), contributing to the highest IHC scores for wound healing-related signaling molecules.

**Fig. 9:**
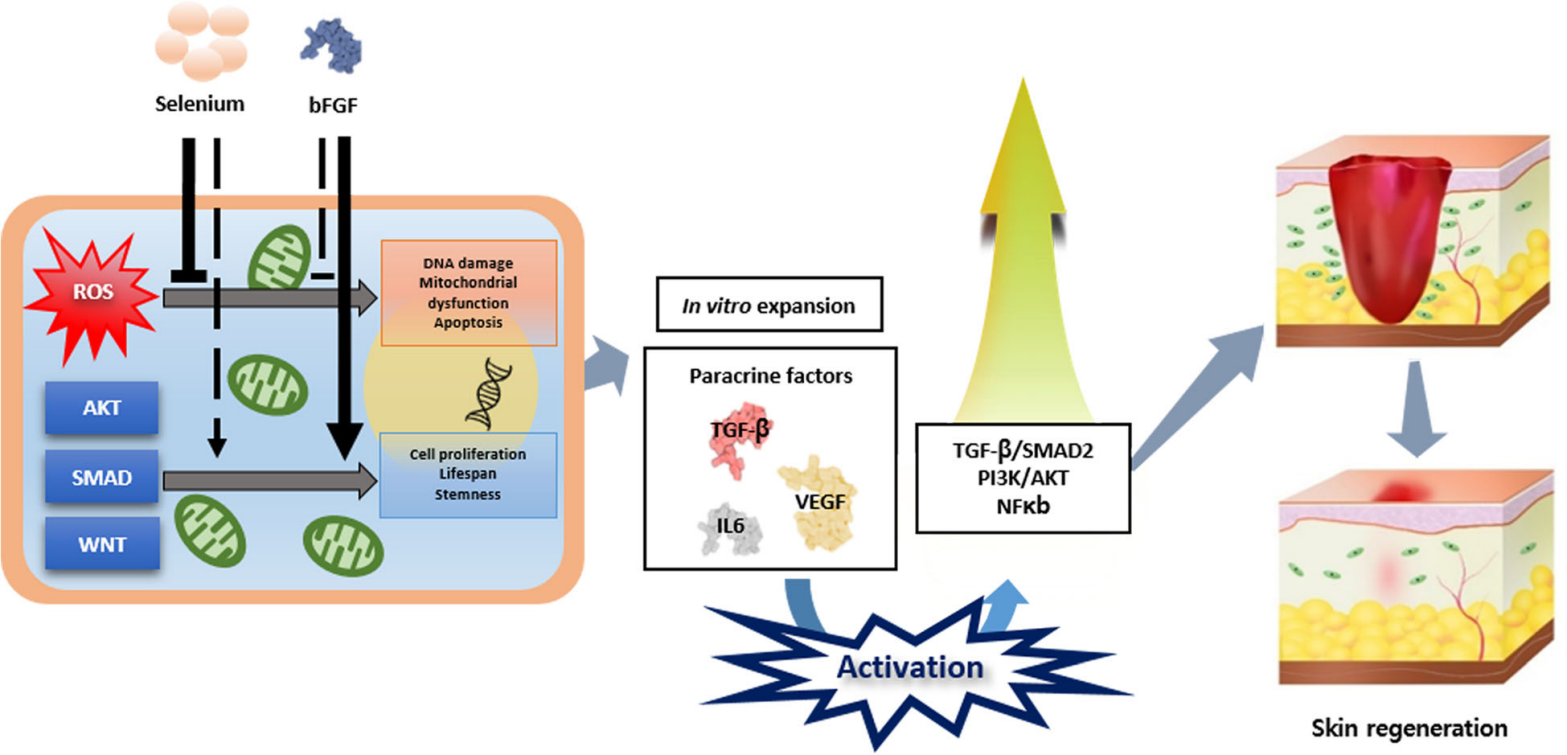
Additive effect of bFGF and selenium on the expansion and paracrine action of human amniotic fluid-derived mesenchymal stem cells

In conclusion, this work clearly indicates that selenium plays a critical role as a ROS scavenger in in vitro expansion of AF-MSCs and long-term maintenance of their multipotency and, when administered together with bFGF, exerts a valuable synergistic effect by inhibiting intracellular ROS accumulation, which otherwise leads to cellular senescence and damage. Regarding the molecular mechanisms important for wound healing and tissue regeneration, bFGF- and selenium-treated AF-MSCs activated several signaling pathways, such as ERK 1/2, AKT, Smad, and NFκB, thereby inducing intensive secretion of growth factors, including TGF-β, VEGF, and IL-6. Furthermore, the CMs derived from AF-MSCs exposed to bFGF and selenium enhanced fibroblast proliferation and migration in vitro and wound closure in vivo. As expected, the degree to which AF-MSC-CMs exert paracrine effects on the in vitro*/*in vivo models exceptionally increased when bFGF and selenium were combined (Fig. 9). These results may provide information on the in vitro expansion of MSCs and the therapeutic efficacy of MSC-CMs, and they further contribute to a better understanding of the paracrine mechanisms of MSCs, especially as they relate to ROS-dependent cellular and molecular signaling.

## Conclusion

The present study suggests that antioxidants such as selenium could be considered as an essential supplement for the paracrine effects of AF-MSCs or, at least, an effective alternative to other expensive supplements, such as growth factors and cytokines.

## Additional files

Additional file 1: Primer sequences used in reverse-transcription PCR and real-time PCR. (PDF 118 kb)

Additional file 2: Antibodies used for Western blotting and immunohistochemistry. (PDF 78 kb)

Additional file 3: Karyotype analysis of AF-MSCs (treated with b/−, −/s, or b/s) was performed using the G-banding method. (PDF 60 kb)

## Abbreviations

AF: Amniotic fluid
CM: Conditioned media
ECM: Extracellular matrix
FBS: Fetal bovine serum
IL-6: Interleukin-6
MMP1: Matrix metalloproteinase1
MSC: Mesenchymal stem cell
ROS: Reactive oxygen species
TGF-β: Transforming growth factor-β
TNF: Tumor necrosis factor
VEGF: Vascular endothelial growth factor

## References

[1] Falanga V (2005). Wound healing and its impairment in the diabetic foot. Lancet.

[2] Tracy LE, Minasian RA, Caterson EJ (2016). Extracellular matrix and dermal fibroblast function in the healing wound. Adv Wound Care.

[3] Tsai MS, Hwang SM, Tsai YL, Cheng FC, Lee JL, Chang YJ (2006). Clonal amniotic fluid-derived stem cells express characteristics of both mesenchymal and neural stem cells. Biol Reprod.

[4] De Coppi P, Bartsch G, Siddiqui MM, Xu T, Santos CC, Perin L (2007). Isolation of amniotic stem cell lines with potential for therapy. Nat Biotechnol.

[5] Uccelli A, Moretta L, Pistoia V (2008). Mesenchymal stem cells in health and disease. Nat Rev Immunol.

[6] Fauza D (2004). Amniotic fluid and placental stem cells. Best Pract Res Clin Obstet Gynaecol.

[7] Jackson WM, Nesti LJ, Tuan RS (2012). Concise review: clinical translation of wound healing therapies based on mesenchymal stem cells. Stem Cells Transl Med.

[8] Shohara R, Yamamoto A, Takikawa S, Iwase A, Hibi H, Kikkawa F (2012). Mesenchymal stromal cells of human umbilical cord Wharton’s jelly accelerate wound healing by paracrine mechanisms. Cytotherapy.

[9] Chen L, Tredget EE, Wu PY, Wu Y (2008). Paracrine factors of mesenchymal stem cells recruit macrophages and endothelial lineage cells and enhance wound healing. PLoS One.

[10] Walter MN, Wright KT, Fuller HR, MacNeil S, Johnson WE (2010). Mesenchymal stem cell-conditioned medium accelerates skin wound healing: an in vitro study of fibroblast and keratinocyte scratch assays. Exp Cell Res.

[11] Yoon BS, Moon JH, Jun EK, Kim J, Maeng I, Kim JS (2010). Secretory profiles and wound healing effects of human amniotic fluid-derived mesenchymal stem cells. Stem Cells Dev.

[12] Lee EY, Xia Y, Kim WS, Kim MH, Kim TH, Kim KJ (2009). Hypoxia-enhanced wound-healing function of adipose-derived stem cells: increase in stem cell proliferation and up-regulation of VEGF and bFGF. Wound Repair Regen.

[13] Tsai MS, Lee JL, Chang YJ, Hwang SM (2004). Isolation of human multipotent mesenchymal stem cells from second-trimester amniotic fluid using a novel two-stage culture protocol. Hum Reprod.

[14] Roth M, Spaniol K, Kordes C, Schwarz S, Mertsch S, Haussinger D (2015). The influence of oxygen on the proliferative capacity and differentiation potential of lacrimal gland-derived mesenchymal stem cells. Invest Ophthalmol Vis Sci.

[15] Sui BD, Hu CH, Zheng CX, Jin Y (2016). Microenvironmental views on mesenchymal stem cell differentiation in aging. J Dent Res.

[16] Devasagayam TP, Tilak JC, Boloor KK, Sane KS, Ghaskadbi SS, Lele RD (2004). Free radicals and antioxidants in human health: current status and future prospects. J Assoc Physicians India.

[17] Imhoff BR, Hansen JM (2011). Differential redox potential profiles during adipogenesis and osteogenesis. Cell Mol Biol Lett.

[18] Kim KS, Choi HW, Yoon HE, Kim IY (2010). Reactive oxygen species generated by NADPH oxidase 2 and 4 are required for chondrogenic differentiation. J Biol Chem.

[19] Helmy MH, Ismail SS, Fayed H, El-Bassiouni EA (2000). Effect of selenium supplementation on the activities of glutathione metabolizing enzymes in human hepatoma Hep G2 cell line. Toxicology.

[20] Saito Y, Yoshida Y, Akazawa T, Takahashi K, Niki E (2003). Cell death caused by selenium deficiency and protective effect of antioxidants. J Biol Chem.

[21] Frost DV, Lish PM (1975). Selenium in biology. Annu Rev Pharmacol.

[22] Oh SH, Ganther HE, Hoekstra WG (1974). Selenium as a component of glutathione peroxidase isolated from ovine erythrocytes. Biochemistry.

[23] Mehta SL, Kumari S, Mendelev N, Li PA (2012). Selenium preserves mitochondrial function, stimulates mitochondrial biogenesis, and reduces infarct volume after focal cerebral ischemia. BMC Neurosci.

[24] Stewart AA, Byron CR, Pondenis H, Stewart MC (2007). Effect of fibroblast growth factor-2 on equine mesenchymal stem cell monolayer expansion and chondrogenesis. Am J Vet Res.

[25] Farre J, Roura S, Prat-Vidal C, Soler-Botija C, Llach A, Molina CE (2007). FGF-4 increases in vitro expansion rate of human adult bone marrow-derived mesenchymal stem cells. Growth Factors.

[26] Yanada S, Ochi M, Kojima K, Sharman P, Yasunaga Y, Hiyama E (2006). Possibility of selection of chondrogenic progenitor cells by telomere length in FGF-2-expanded mesenchymal stromal cells. Cell Prolif.

[27] Tarte K, Gaillard J, Lataillade JJ, Fouillard L, Becker M, Mossafa H (2010). Clinical-grade production of human mesenchymal stromal cells: occurrence of aneuploidy without transformation. Blood.

[28] Jun EK, Zhang Q, Yoon BS, Moon JH, Lee G, Park G (2014). Hypoxic conditioned medium from human amniotic fluid-derived mesenchymal stem cells accelerates skin wound healing through TGF-beta/SMAD2 and PI3K/Akt pathways. Int J Mol Sci.

[29] Moon JH, Kwak SS, Park G, Jung HY, Yoon BS, Park J (2008). Isolation and characterization of multipotent human keloid-derived mesenchymal-like stem cells. Stem Cells Dev.

[30] Kern S, Eichler H, Stoeve J, Kluter H, Bieback K (2006). Comparative analysis of mesenchymal stem cells from bone marrow, umbilical cord blood, or adipose tissue. Stem Cells.

[31] Kho Y, Kim S, Yoon BS, Moon JH, Kim B, Kwak S (2008). Induction of serum amyloid A genes is associated with growth and apoptosis of HC11 mammary epithelial cells. Biosci Biotechnol Biochem.

[32] Miller GE, Chen E (2006). Life stress and diminished expression of genes encoding glucocorticoid receptor and beta2-adrenergic receptor in children with asthma. Proc Natl Acad Sci U S A.

[33] Galiano RD, Michaels JT, Dobryansky M, Levine JP, Gurtner GC (2004). Quantitative and reproducible murine model of excisional wound healing. Wound Repair Regen.

[34] McDonald JW, Pilgram TK (1999). Nuclear expression of p53, p21 and cyclin D1 is increased in bronchioloalveolar carcinoma. Histopathology.

[35] Nekanti U, Mohanty L, Venugopal P, Balasubramanian S, Totey S, Ta M (2010). Optimization and scale-up of Wharton’s jelly-derived mesenchymal stem cells for clinical applications. Stem Cell Res.

[36] Campisi J, Robert L (2014). Cell senescence: role in aging and age-related diseases. Interdiscip Top Gerontol.

[37] Coutu DL, Galipeau J (2011). Roles of FGF signaling in stem cell self-renewal, senescence and aging. Aging.

[38] Bianchi G, Banfi A, Mastrogiacomo M, Notaro R, Luzzatto L, Cancedda R (2003). Ex vivo enrichment of mesenchymal cell progenitors by fibroblast growth factor 2. Exp Cell Res.

[39] Pricola KL, Kuhn NZ, Haleem-Smith H, Song Y, Tuan RS (2009). Interleukin-6 maintains bone marrow-derived mesenchymal stem cell stemness by an ERK1/2-dependent mechanism. J Cell Biochem.

[40] Kumar A, Salimath BP, Stark GB, Finkenzeller G (2010). Platelet-derived growth factor receptor signaling is not involved in osteogenic differentiation of human mesenchymal stem cells. Tissue Eng A.

[41] Paradies G, Petrosillo G, Paradies V, Ruggiero FM (2010). Oxidative stress, mitochondrial bioenergetics, and cardiolipin in aging. Free Radic Biol Med.

[42] Passos JF, Saretzki G, Ahmed S, Nelson G, Richter T, Peters H (2007). Mitochondrial dysfunction accounts for the stochastic heterogeneity in telomere-dependent senescence. PLoS Biol.

[43] Hu Y, Zhang Y, Tian K, Xun C, Wang S, Lv D (2016). Effects of nerve growth factor and basic fibroblast growth factor dual gene modification on rat bone marrow mesenchymal stem cell differentiation into neuron-like cells in vitro. Mol Med Rep.

[44] Shalini Vellasamy SV, George E, Ramasamy R (2016). Basic fibroblast growth factor enhances the expansion and secretory profile of human placenta-derived mesenchymal stem cells. Malays J Med Health Sci.

[45] Battin EE, Brumaghim JL (2009). Antioxidant activity of sulfur and selenium: a review of reactive oxygen species scavenging, glutathione peroxidase, and metal-binding antioxidant mechanisms. Cell Biochem Biophys.

[46] Hosseinzadeh Anvar Leila, Hosseini-Asl Saeid, Mohammadzadeh-Vardin Mohammad, Sagha Mohsen (2017). The Telomerase Activity of Selenium-Induced Human Umbilical Cord Mesenchymal Stem Cells Is Associated with Different Levels of c-Myc and p53 Expression. DNA and Cell Biology.

[47] Valadbeygi A, Naji T, Pirnia A, Gholami M (2016). Supplementation freeze-thawed media with selenium protect adipose-derived mesenchymal stem cells from freeze-thawed induced injury. Cryobiology.

[48] Shi H, Cheng Y, Ye J, Cai P, Zhang J, Li R (2015). bFGF promotes the migration of human dermal fibroblasts under diabetic conditions through reactive oxygen species production via the PI3K/Akt-Rac1- JNK pathways. Int J Biol Sci.

[49] Kanazawa S, Fujiwara T, Matsuzaki S, Shingaki K, Taniguchi M, Miyata S (2010). bFGF regulates PI3-kinase-Rac1-JNK pathway and promotes fibroblast migration in wound healing. PLoS One.

[50] Yang Y, Xia T, Zhi W, Wei L, Weng J, Zhang C (2011). Promotion of skin regeneration in diabetic rats by electrospun core-sheath fibers loaded with basic fibroblast growth factor. Biomaterials.

[51] Zhu ZX, Cai WH, Wang T, Ye HB, Zhu YT, Chi LS (2015). bFGF-regulating MAPKs are involved in high glucose-mediated ROS production and delay of vascular endothelial cell migration. PLoS One.

[52] Roberge S, Roussel J, Andersson DC, Meli AC, Vidal B, Blandel F (2014). TNF-alpha-mediated caspase-8 activation induces ROS production and TRPM2 activation in adult ventricular myocytes. Cardiovasc Res.

[53] Bao C, Guo J, Zheng M, Chen Y, Lin G, Hu M. Enhancement of the survival of engrafted mesenchymal stem cells in the ischemic heart by TNFR gene transfection. Biochem Cell Biol 2010;88:629–634. 10.1139/O10-018 20651834

[54] Geissler S, Textor M, Kuhnisch J, Konnig D, Klein O, Ode A (2012). Functional comparison of chronological and in vitro aging: differential role of the cytoskeleton and mitochondria in mesenchymal stromal cells. PLoS One.

[55] Barrientos S, Stojadinovic O, Golinko MS, Brem H, Tomic-Canic M (2008). Growth factors and cytokines in wound healing. Wound Repair Regen.

[56] Lin ZQ, Kondo T, Ishida Y, Takayasu T, Mukaida N (2003). Essential involvement of IL-6 in the skin wound-healing process as evidenced by delayed wound healing in IL-6-deficient mice. J Leukoc Biol.

[57] Frantz C, Stewart KM, Weaver VM (2010). The extracellular matrix at a glance. J Cell Sci.

[58] Bainbridge P (2013). Wound healing and the role of fibroblasts. J Wound Care.

[59] Schiller M, Javelaud D, Mauviel A (2004). TGF-beta-induced SMAD signaling and gene regulation: consequences for extracellular matrix remodeling and wound healing. J Dermatol Sci.

[60] Liu RM, Desai LP (2015). Reciprocal regulation of TGF-beta and reactive oxygen species: a perverse cycle for fibrosis. Redox Biol.

[61] De Miguel MP, Fuentes-Julian S, Blazquez-Martinez A, Pascual CY, Aller MA, Arias J (2012). Immunosuppressive properties of mesenchymal stem cells: advances and applications. Curr Mol Med.

