# Supplementary material for: Additive effect of bFGF and selenium on expansion and paracrine action of human amniotic fluid-derived mesenchymal stem cells
Source: Stem Cell Res Ther. 2018 Nov 8;9:293. doi: 10.1186/s13287-018-1058-z (PMC6225588; doi:10.1186/s13287-018-1058-z)
Supplement: Supplementary file 1 — Primer sequences used in reverse-transcription PCR and real-time PCR. (PDF 118 kb) [file 13287_2018_1058_MOESM1_ESM.pdf]

Gene	Accesion no.	Primer sequence (5’-3’)	Anealing temperature (℃)	Product size (dp)
VEGF	NM_001204385.1	F-CTACCTCCACCATGCCAAGT<br>R-GCAGTAGCTGCGCTGATAGA	62	109
TGF-β	NM_000660.5	F-CATGGCATGAACCGGCCTTT<br>R-CGAGGTCCTTGCGGAAGTCAAT	62	176
IL6	NM_001318095.1	F-GCCAGAGCTGTGCAGATGAG<br>R-TTGTCATGTCCTGCAGCCAC	62	154
Collagen 3	NM_000090.3	F-CTGAAATTCTGCCATCCTGAAC<br>R-GGATTGCCGTAGCTAAACTGAA	62	236
MMP1	NM_002421.2	F-TTGAGAAAGCCTTCCAACTCTG<br>R-CCGCAACACGATGTAAGTTGTA	62	250
SPP1	NM_0010400060.1	F-CTGGAAGTTCTGAGGAAAAGCA<br>R-TCAGGGTACTGGATGTCAGGTC	62	401
Syndecan 4	NM_002999.2	F-CCTAGAAGGCCGATACTTCTCC<br>R-ACCTTGTTGGACACATCCTCAC	62	292
GAPDH	NM_002046	F-GTGGTCTCCTCTGACTTCAACA<br>R-CTCTTCCTCTTGTGCTCTTGCT	62	211
