# Supplementary material for: Additive effect of bFGF and selenium on expansion and paracrine action of human amniotic fluid-derived mesenchymal stem cells
Source: Stem Cell Res Ther. 2018 Nov 8;9:293. doi: 10.1186/s13287-018-1058-z (PMC6225588; doi:10.1186/s13287-018-1058-z)
Supplement: Supplementary file 2 — Antibodies used for Western blotting and immunohistochemistry. (PDF 78 kb) [file 13287_2018_1058_MOESM2_ESM.pdf]

**Antibody**	**Company**	**Host**
Akt	Cell signaling	Rabbit
p-Akt	Cell signaling	Rabbit
PI3K	Cell signaling	Rabbit
p-MEK	Cell signaling	Rabbit
MEK	Cell signaling	Rabbit
p-ERK	Cell signaling	Rabbit
ERK	Santa Cruz	Goat
p-SMAD2	Cell signaling	Rabbit
p-SMAD3	Cell signaling	Rabbit
SMAD2/3	Cell signaling	Rabbit
aP2	Abcam	Rabbit
PPARγ	Abcam	Mouse
LPL	Abcam	Mouse
Osteocalcin	Abcam	Rabbit
Osteopontin	Abcam	Rabbit
Aggrican	Abcam	Rabbit
CollagenII	Millipore	Mouse
TGF-β1	Cell signaling	Rabbit
NF-kB	Cell signaling	Rabbit
p-NFkB	Cell signaling	Rabbit
Involucrin	Thermofisher scientific	Mouse
Fibronectin	Abcam	Mouse
Vitronectin	Millipore	Mouse
Syndecan2	Millipore	Mouse
Elastin	Santa Cruz	Mouse
β-Actin	Sigma Aldrich	Rabbit
Tubulin	Sigma Aldrich	Mouse
