# Supplementary figures and images for: Additive effect of bFGF and selenium on expansion and paracrine action of human amniotic fluid-derived mesenchymal stem cells
Source: Stem Cell Res Ther. 2018 Nov 8;9:293. doi: 10.1186/s13287-018-1058-z (PMC6225588; doi:10.1186/s13287-018-1058-z)

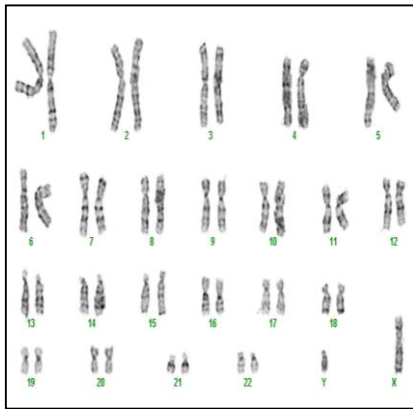

**(b/-)**

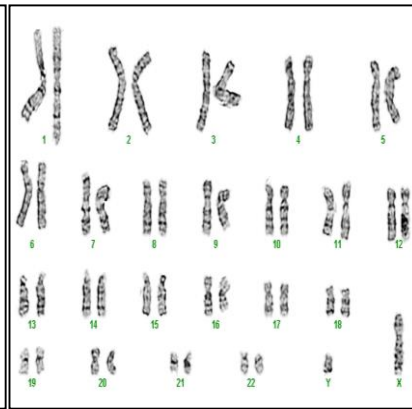

**(-/s)**

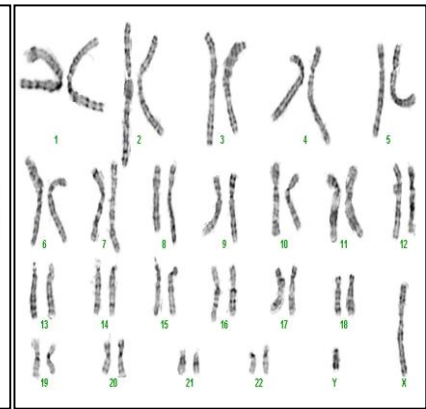

**(b/s)**

Supplement: Supplementary file 3 — Karyotype analysis of AF-MSCs (treated with b/−, −/s, or b/s) was performed using the G-banding method. (PDF 60 kb) [file 13287_2018_1058_MOESM3_ESM.pdf]
